# "Drunk or Dying" Cases

**Published:** 1903-02-07

**Authors:** 


### "DRUNK OR DYING" CASES.

Amongst the most pressing questions which affect the general hospitals of London is that of "drunk or dying" cases, like the recent one of Rose Askew at St. Mary's. It is one of many nearly identical cases which have happened, been inquired into by the coroner, and reported in the papers during the last 30 years. How and why are such scandals permitted to occur time after time? With whom lies the responsibility, and what is the remedy? These are the points which it is essential to have clearly in mind definitely and finally. When this has been done, and the responsibility has been fixed, there can be little doubt that those to whom the management of the different London general hospitals is entrusted will make it their duty forthwith to arrange that in the future such mistakes cannot recur. Not only does an accident, like the one we have referred to, by receiving wide publicity in the Press, bring discredit upon the particular hospital implicated, but it lowers the prestige of all the general hospitals in London, while confidence is lowered in the medical men connected with them. The ultimate result must almost certainly have a prejudicial effect upon the subscriptions which are given to the hospitals.

### The St. Mary's Case.

Briefly stated, the facts of the St. Mary's case are these:—Rose Askew, aged 41, while under the influence of drink, in attempting to cross the street, was knocked down by a passing cab. She was picked up and taken to St. Mary's Hospital, where the house-surgeon dressed some slight wounds, and, evidently in some doubt as to the extent of the injuries she had received, kept her for some time under observation in the waiting-room. He then ordered her removal to the infirmary, where she subsequently died as a result of the injuries she had received at the time of the accident. Since no provision is made at St. Mary's Hospital for keeping a case like this under observation, say for 24 hours, unless the patient is admitted into a general ward—a proceeding which is obviously unwarrantable in the interests of the other patients—it seems pretty clear that the house surgeon, in sending the woman to the infirmary, adopted the only course which was open to him. Had he allowed an alcoholic, filthy person to be admitted into a general ward his action would most probably have been resented by patients, nurses, and hospital authorities alike. At the best managed general hospitals in London such difficulties do not arise, because at these institutions accommodation is provided for keeping these doubtful cases under observation for such time as is needful to make plain the diagnosis. It is to be hoped that the rest of the London general hospitals will immediately take some steps to provide beds for these patients in emergency wards, where doubtful cases may be detained for, say, 24 hours, for the purpose of avoiding in future the reproach of having sent away from their doors patients in a dying condition.

### Responsibility of House-Surgeons.

From the moment when any such "Drunk or Dying" case is brought into the casualty room of a general hospital, the house-surgeon on duty becomes responsible for the patient, and his responsibility continues until the case has been seen by a member of the visiting staff of the hospital, or has been transferred to the care of some other medical man, whether at an infirmary or elsewhere. But the house-surgeon's responsibility is limited entirely by the means which have been placed at his disposal by the hospital managers. The house-surgeon in a London hospital is usually not in office more than 12 months. This involves a constant change, and every six or 12 months a new man comes on who more often than not has had but little, if any, previous experience in positions of such responsibility. Consequently, when a patient is sent away from a general hospital to die in a police cell or at an infirmary, it is not uncommon for the fault to be laid at the door of the house-surgeon's inexperience, and this is the more liable to come about since the hospital authorities readily repudiate any share in the mistake. Whereas, relatively inexperienced and young as house-surgeons often are, there is not one of them but knows full well that neither himself, or any one else, however experienced he may be, can always diagnose at once whether a patient is only drunk or whether he is dangerously ill from some injury or lesion. We have said in dealing with the St. Mary's case that the house-surgeon could not keep the patient in the hospital, because the authorities had failed to provide the necessary accommodation. He was compelled to send her away, whether to the infirmary or the police station. And to us it seems clear therefore that with the hospital managers, *i.e.* the committee, alone rest the responsibility, and that with them it will remain until they have provided the necessary accommodation and nursing which such cases require.

Wanted : Casualty Beds.

The twelve great general hospitals of London are the London, Guy's, St. Bart.'s, St. Thomas's, St George's, Middlesex, St. Mary's, King's College, University College, Westminster, Charing Cross, and the Royal Free Hospital. At only four of the twelve has any attempt whatever been made to provide temporary beds for these doubtful cases. They are Charing Cross, St. Bartholomew's, University College, and St. George's Hospitals. That accommodation is as follows :—Charing Cross Hospital has a special ward containing three beds set apart for these doubtful cases, which can remain there for 24 hours if necessary. The emergency case is nursed from an adjacent general ward, and is reported to be worked well and satisfactorily. St. Bartholomew's has a surgery ward which fulfils the same purposes. At University College Hospital are some surgery beds which may be occupied from 8 p.m. till 8 a.m. They are not open for use during the daytime. St. George's can scarcely be said to have provided adequate accommodation of this kind, but they have three beds in the casualty department, separable from the main rooms only by screens, but which they say are available for use if required. It does not appear that they are so often required at St. George's as they are elsewhere. Some of the remaining general hospitals have expressed the intention of instituting these necessary casualty beds.

What the Public Demands.

We have shown what is the cause of these "drunk or dying" scandals, what the remedy is and with whom the responsibility rests. We trust that the remedy will be provided by the managers of every general hospital in London without delay. The London public demands that its general hospitals shall be so conducted that no unnecessary suffering shall be entailed upon the patients who seek relief, and that every facility shall be provided for the medical officers to carry on their work to the best advantage of patients and hospital alike. Moreover the public has shown an increasing power of discrimination between the different claims of the various charities it supports; and that the best claim that any charitable institution can place before the public is that of complete efficiency in every detail and branch of the work which it has undertaken.

